# Systematic Review and Meta-Analysis of Renin–Angiotensin–Aldosterone System Blocker Effects on the Development of Cardiovascular Disease in Patients With Chronic Kidney Disease

**DOI:** 10.3389/fphar.2021.662544

**Published:** 2021-07-02

**Authors:** Katsunori Yanai, Kenichi Ishibashi, Yoshiyuki Morishita

**Affiliations:** ^1^ First Department of Integrated Medicine, Division of Nephrology, Saitama Medical Center, Jichi Medical University, Saitama, Japan; ^2^ Department of Medical Physiology, Meiji Pharmaceutical University, Tokyo, Japan

**Keywords:** renin-angiotensin-aldosterone system blocker, cardiovascular disease, chronic kidney disease, pre-dialysis, hemodialysis, peritoneal dialysis, systematic review, meta-analysis

## Abstract

**Background:** Cardiovascular events are one of the most serious complications that increase the risk of mortality and morbidity in pre-dialysis and on-dialysis chronic kidney disease (CKD) patients. Activation of the renin–angiotensin–aldosterone system (RAAS) is considered to contribute to the development of cardiovascular events in these populations. Therefore, several kinds of RAAS blockers have been frequently prescribed to prevent cardiovascular events in patients with CKD; however, their effectiveness remains controversial. This systematic review focuses on whether RAAS blockers prevent cardiovascular events in patients with CKD.

**Method:** PubMed were searched to retrieve reference lists of eligible trials and related reviews. Randomized prospective controlled trials that investigated the effects on cardiovascular events in CKD patients that were published in English from 2010 to 2020 were included.

**Results:** Among 167 identified studies, 11 eligible studies (*n* = 8,322 subjects) were included in the meta-analysis. The meta-analysis showed that RAAS blockers significantly reduced cardiovascular events in on-dialysis patients with CKD [three studies; odds ratio (OR), 0.52; 95% confidence interval (CI), 0.36 to 0.74; *p* = 0.0003], but there was no significant difference in pre-dialysis patients with CKD because of the heterogeneity in each study (eight studies). We also investigated the effects of each kind of RAAS blocker on cardiovascular events in CKD patients. Among the RAAS blockers, mineralocorticoid receptor antagonists significantly decreased cardiovascular events in pre-dialysis or on-dialysis patients with CKD (four studies; OR, 0.60; 95%CI, 0.50 to 0.73, *p* < 0.0001). However, angiotensin receptor blockers did not show significant effects (four studies; OR, 0.65; 95%CI, 0.42 to 1.01; *p* = 0.0529). The effects of angiotensin converting enzyme inhibitors and direct renin inhibitors on cardiovascular events in patients with CKD could not be analyzed because there were too few studies.

**Conclusion:** Mineralocorticoid receptor antagonists may decrease cardiovascular events in pre-dialysis or on-dialysis patients with CKD.

## Introduction

Cardiovascular events are one of the most serious complications that increase the risk of mortality and morbidity in chronic kidney disease (CKD) patients who are undergoing pre-dialysis, hemodialysis, or peritoneal dialysis (Kim-Mitsuyama et al., 2018; Tonelli et al., 2019). Activation of the renin–angiotensin–aldosterone system (RAAS) is considered to be an important factor that contributes to the development of cardiovascular disease in patents with CKD (Liu et al., 2014). Therefore, several types of RAAS blockers including angiotensin converting enzyme inhibitors (ACEIs), angiotensin receptor blockers (ARBs), mineralocorticoid receptor antagonists (MRAs), and direct renin inhibitors (DRIs) have been frequently prescribed, and they are expected to prevent cardiovascular events and have reno-protective effects in patients with CKD. However, their protective effects in cardiovascular events in this population remain controversial (Xie et al., 2016). Additionally, different types of RAAS blockers may have different effects on reducing cardiovascular events in patients with CKD. To address these clinical questions, this systematic review focuses on whether each RAAS blocker prevents cardiovascular events in pre-dialysis or on-dialysis patients with CKD.

### The Effects of Each Class of Renin–Angiotensin–Aldosterone System Blockers

#### Angiotensin Receptor Blockers

ARBs bind angiotensin receptor-1 and inhibit angiotensin II from binding to angiotensin receptor-1 (Pang et al., 2012). They then suppress vasoconstriction resulting in a decrease in blood pressure (Pang et al., 2012). ARBs act directly on vascular smooth muscle and suppress aldosterone secretion, thereby preventing sodium accumulation and lowering blood pressure, which leads to inhibition of fibrosis of heart and kidney (Hara et al., 2017; Isobe-Sasaki et al., 2017; Zhang et al., 2017).

#### Angiotensin Converting Enzyme Inhibitors

ACEIs activate angiotensin converting enzyme on the vascular endothelial cell membrane. They prevent the conversion of angiotensin I into angiotensin II by inhibiting angiotensin converting enzyme and then suppress vasoconstriction, which results in deceasing blood pressure (Ali et al., 2019; Park et al., 2019).

#### Mineralocorticoid Receptor Antagonists

MRAs show antihypertensive effects by competitively binding to mineralocorticoid receptors on the distal tubules and collecting ducts of the kidney and inhibiting the effects of mineralocorticoids (Farman and Rafestin-Oblin, 2001). MRAs excrete sodium and absorb potassium and hydrogen, resulting in a reduction in the volume of circulating and extracellular fluids and thereby a reduction in blood pressure (Sato et al., 2003) and an improvement in edema.

#### Direct Renin Inhibitors

DRIs act upon renin, which converts angiotensinogen into angiotensin-1. They inhibit plasma renin activity, which causes a decrease in blood pressure (Morishita and Kusano, 2013).

## Methods

### Literature Search

**FIGURE 1:**
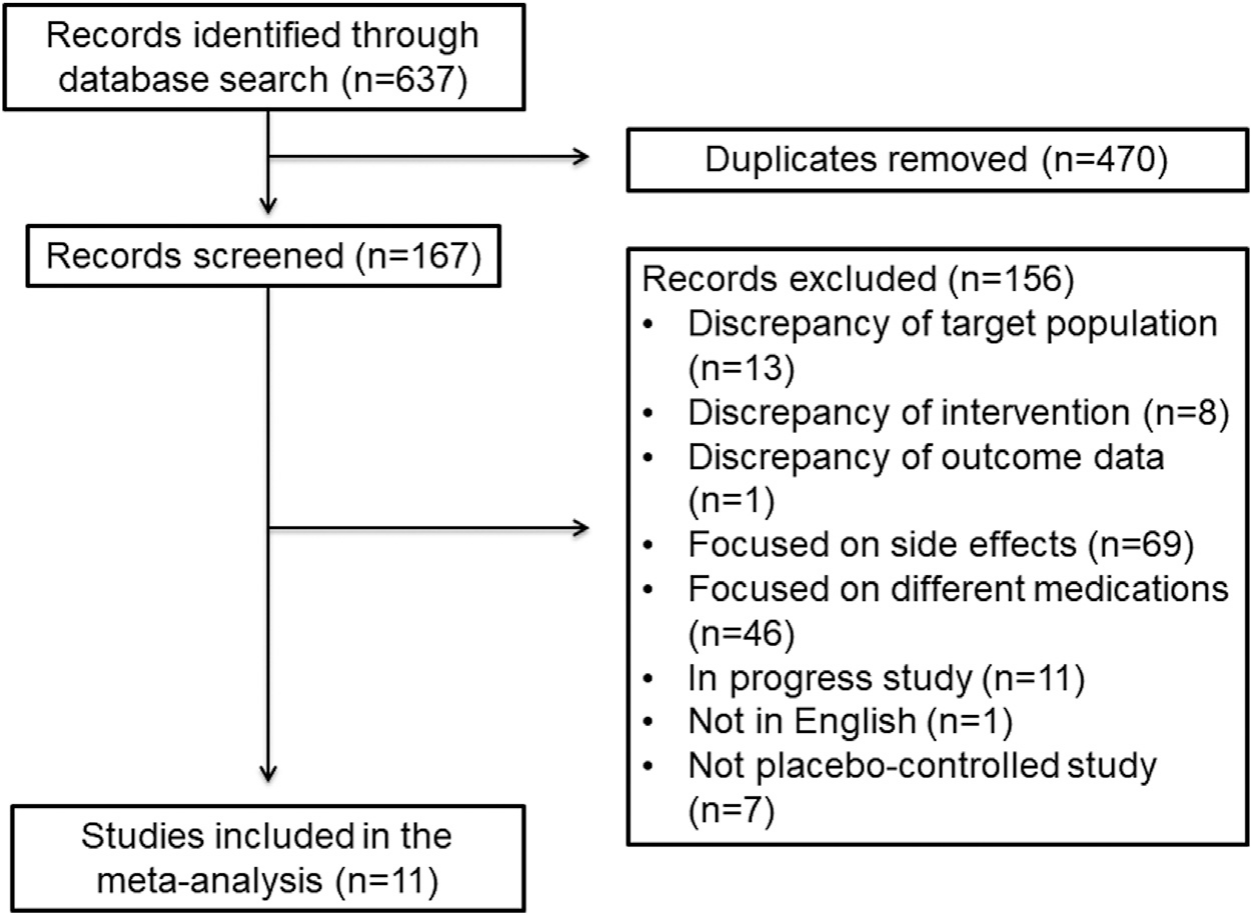
Flow diagram of this systemic review and meta-analysis

We searched for clinical studies that were published in English in the PubMed database from 2010 to 2020. A literature search was conducted between November 9 and 16, 2020. For each term of “chronic kidney disease,” “hemodialysis,” and “peritoneal dialysis,” we searched by connecting with terms including “cardiovascular disease,” “heart failure,” “heart attack,” and “renin angiotensin aldosterone,” “angiotensin receptor blocker,” “ARB,” “angiotensin converting enzyme inhibitor,” “ACEI,” “mineralocorticoid receptor antagonist,” “direct renin inhibitor” as listed in Supplementary Table S1. We limited the article type to randomized controlled studies. The studies’ eligibility was carefully checked for inclusion in accordance with Preferred Reporting Items for Systematic and Meta-Analyses (PRISMA) guidelines (Figure 1; Shamseer et al., 2015). The inclusion criteria for the studies were as follows: 1) the study reported the effects of RAAS blockers on cardiovascular events such as heart failure, stroke, myocardial infarction, and unstable angina in patients with CKD; and 2) the study was published as a full-text journal article in English. Exclusion criteria were as follows: 1) the effects of RAAS blockers on the cardiovascular events in patients with CKD were not mentioned; 2) there was no description of sample settings; 3) the study focused on side effects; 4) the study was ongoing; 5) the study was not published in English; 6) the study was not a placebo-controlled study; 7) there was no detailed description of outcome data; and 8) other cardioprotective drugs, such as diuretics and beta-blockers, were prescribed as an intervention (Bristow, 2011; Pugh et al., 2019). We also evaluated references that seems to be important from guidelines.

### Statistical Methods

The main objective of this study was to access the incidence of cardiovascular diseases in CKD patients with RAAS blocker treatment across different CKD types and to compare the relative risk of cardiovascular diseases between RAAS blockers and placebo. We calculated the incidence of cardiovascular diseases from the data that were available in each study. A meta-analysis was conducted with R software (version 4.0.3) (R Foundation for Statistical Computing, Vienna, Austria) using the Mantel–Haenszel and DerSimonian–Laird methods. A *p* value less than 0.05 was considered to represent statistical significance. Bonferroni adjustment for multiple testing in meta-analysis produced a rejection *p*-value of 0.05 divided by the total number of outcomes. Incidence rates for each study are displayed in forest plots with the estimated 95% confidence intervals (CIs). The relative risk and corresponding 95% CIs were also calculated for patients who were treated with a RAAS blocker compared with placebo. The statistical heterogeneity among the selected studies was verified using the Cochrane Q statistic and the *I* ^2^ statistic. If there was no statistically significant heterogeneity (*p* > 0.05 or *I* ^2^ < 40%) among the results of the included trials, the pooled estimate was calculated based on the fixed-effects model. If significant heterogeneity (*p* < 0.05 or *I* ^2^ > 40%) was observed in the analysis, a random-effects model was used for the meta-analysis. We determined beneficial effects of RAAS blockers for cardiovascular diseases if the results of the meta-analysis showed *p*-value was below 0.05, 95% CI was below 1.00 (did not cross 1.00), and no heterogeneity of each study was observed using the Cochrane Q statistic, the *I* ^2^ statistic and Bonferroni correction analysis (Grover and Kukreti, 2013). Funnel plots were generated to visually assess asymmetry and potential publication bias, along with the Egger’s test.

## Results

### Search Results

**FIGURE 2:**
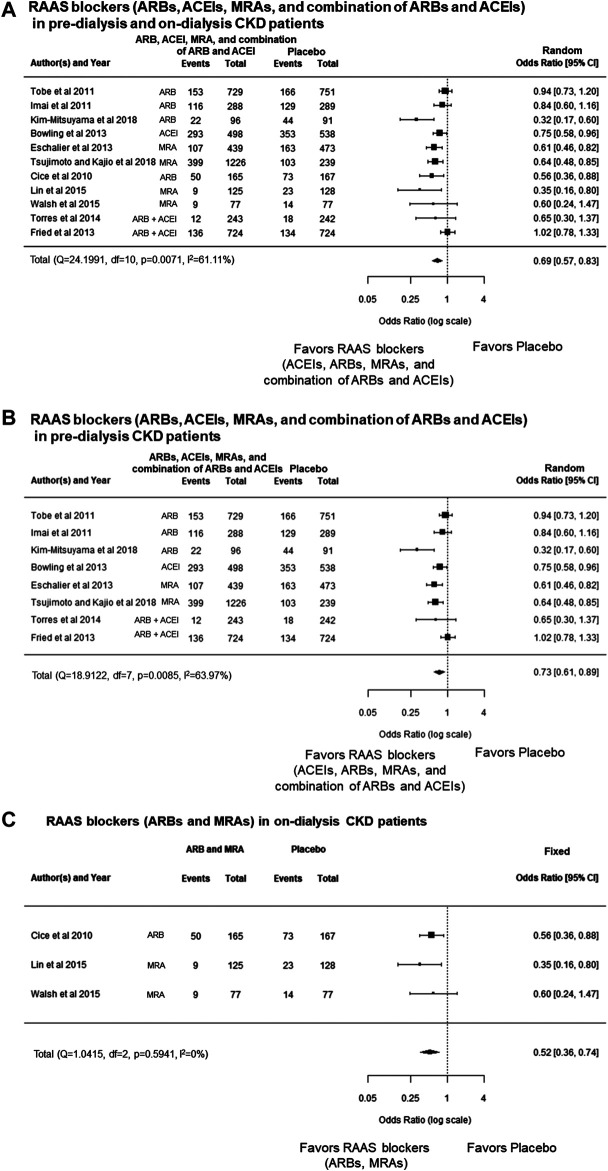
**(A)** Forest plot describing a comparison of the incidence of cardiovascular events between RAAS blockers (ARBs, ACEIs, MRAs, and the combination of ARBs and ACEIs) and placebo in pre-dialysis and on-dialysis patients with CKD. **(B)** Forest plot describing a comparison of the incidence of cardiovascular events between RAAS blockers (ARBs, ACEIs, MRAs, and combination of ARBs and ACEIs) and placebo in pre-dialysis patients with CKD. **(C)** Forest plot describing a comparison of the incidence of cardiovascular events between RAAS blockers (ARBs and MRAs) and placebo in on-dialysis patients with CKD. ACEIs, angiotensin converting enzyme inhibitors; ARBs, angiotensin receptor blockers; CI, confidence intervals; CKD, chronic kidney disease; MRAs, mineralocorticoid receptor antagonists; RAAS, renin–angiotensin–aldosterone system

A flow diagram including the study inclusion and exclusion criteria is presented in Figure 2. Computer and manual searches identified 637 publications. After removing duplicates, 167 articles remained, and among them, 156 articles were excluded because they did not meet the study entry criteria. In patients with CKD, hemodialysis, and peritoneal dialysis, there were publications on the use of RAAS blockers, but they were excluded if they were not related to a direct cardiovascular event. After full-text screening, 11 studies (*n* = 8,322 subjects) were included in this systematic review and meta-analysis (ARBs, *n* = 4; ACEIs, *n* = 1; MRAs, *n* = 4; and combination of ARBs and ACEIs, *n* = 2) (Figure 1) (Cice et al., 2010; Imai et al., 2011; Tobe et al., 2011; Bowling et al., 2013; Eschalier et al., 2013; Fried et al., 2013; Torres et al., 2014; Walsh et al., 2015; Lin et al., 2016; Kim-Mitsuyama et al., 2018; Tsujimoto and Kajio, 2018). No studies that met the study inclusion criteria investigated the effects DRIs.

### The Effects of Renin–Angiotensin–Aldosterone System Blockers for Prevention of Cardiovascular Events in Pre-dialysis and On-Dialysis Chronic Kidney Disease Patients

The meta-analysis showed that RAAS blockers (ARBs, ACEIs, MRAs, and combination of ARBs and ACEIs) significantly decreased cardiovascular events compared with the placebo group in pre-dialysis or on-dialysis patients with CKD [odds ratio (OR), 0.69; 95% CI, 0.57 to 0.83, *p* < 0.0001] (Figure 2A). However, heterogeneity among the cohorts was statistically significant (*p* = 0.0071, *I* ^2^ = 61.11%), which remained significant after Bonferroni correction.

In sub-group analysis, categorized pre-dialysis patients with CKD, and on-dialysis patients with CKD. The meta-analysis also showed that RAAS blockers significantly decreased cardiovascular events compared with placebo groups in pre-dialysis patients with CKD (OR, 0.73; 95% CI, 0.61 to 0.89, *p* = 0.0017) (Figure 2B); however, heterogeneity among cohorts was also statistically significant (*p* = 0.0085, *I* ^2^ = 63.97%), which remained significant after Bonferroni correction. The meta-analysis also showed that RAAS blockers significantly decreased cardiovascular events compared with placebo groups on-dialysis patients with CKD (OR, 0.52; 95% CI, 0.36 to 0.74; *p* = 0.0003), and there was no heterogeneity (*p* = 0.5941, *I* ^2^ = 0.0%) (Figure 2C). These results showed that RAAS blockers significantly decreased cardiovascular events in on-dialysis patients with CKD patients; however, these effects were not shown in pre-dialysis patients with CKD because there was heterogeneity among the cohorts.

**FIGURE 3:**
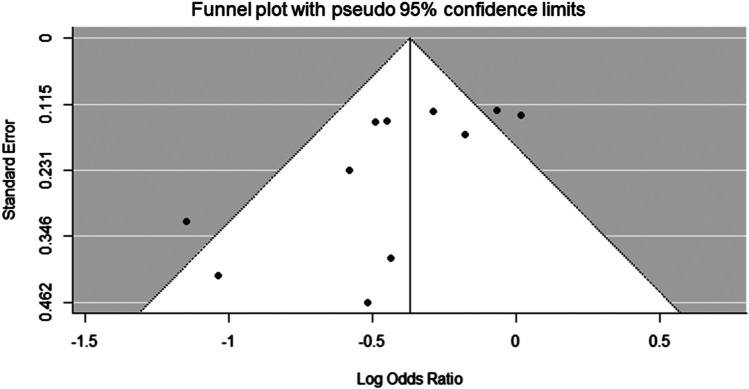
Funnel plot of meta-analysis.

The funnel plot appeared to be asymmetric (Egger’s test, *p* = 0.0091), with some missingness at the lower right portion of the plot suggesting possible publication bias (Figure 3).

### The Effects of Each Class of Renin–Angiotensin–Aldosterone System Blockers (Angiotensin Receptor Blockers, Angiotensin Converting Enzyme Inhibitors, Mineralocorticoid Receptor Antagonists, and Combination of Angiotensin Receptor Blockers and Angiotensin Converting Enzyme Inhibitors) for Prevention of Cardiovascular Events in Pre-dialysis and On-Dialysis Chronic Kidney Disease Patients

In the data base research in pre-dialysis patients with CKD or on-dialysis patients with CKD who took ARBs, three studies in pre-dialysis patients with CKD and on-dialysis patients with CKD, and one study on-dialysis patients with CKD were included in this systematic review and meta-analysis (Cice et al., 2010; Imai et al., 2011; Tobe et al., 2011; Kim-Mitsuyama et al., 2018; Table 1).

**TABLE 1:** Effects of angiotensin receptor blockers on the development of cardiovascular disease in pre-dialysis or on-dialysis patients with CKD.

Class of RAAS blocker	Authors, Year; Reference number	Patients	Study design	Study protocol	Results
ARB	Tobe et al., 2011; 24	N = 1480; eGFR <60 (mL/min/1.73 m^2^) Serum creatinine concentration <3.0 mg/dL	RCT; Multicenter double-blind placebo-controlled clinical trial	Telmisartan 80 mg or placebo once daily; 4–7 years	No improvement in cardiovascular outcomes, including cardiovascular death, myocardial infarction, stroke, and hospitalization for heart failure was found with telmisartan therapy compared with placebo in patients with CKD (p value was not shown).
	Imai et al., 2011; 10	N = 577; Serum creatinine concentration was 1.2–2.5 mg/dL in men and 1.0–2.5 mg/dL in women	RCT; Double-blind placebo-controlled clinical trial; Secondary outcomes	Olmesartan 10–40 mg once daily or placebo; 4 years	No improvement in cardiovascular outcomes, including cardiovascular death, non-fatal stroke except for transient ischemic attack, nonfatal myocardial infarction, hospitalization for unstable angina, hospitalization for heart failure, revascularization of coronary was found with olmesartan therapy compared with placebo in patients with CKD (HR, 0.73; 95% CI, 0.48 to 1.09; p = 0.126).
	Kim-Mitsuyama et al., 2018; 13	N = 187; eGFR <45 (mL/min/1.73 m^2^)	RCT; Multicenter open-label placebo-controlled clinical trial; Secondary outcomes	Olmesartan 20–80 mg once daily or placebo; 3 years	In patients with advanced CKD, olmesartan-based therapy may confer greater benefit in prevention of cardiovascular events than placebo therapy (HR, 0.465; 95% CI, 0.224 to 0.965; p = 0.040).
	Cice et al., 2010; 4	N = 332; Hemodialysis; Chronic heart failure with reduced ejection fraction <40% within 6 months	RCT; Multicenter double-blind placebo-controlled clinical trial	Telmisartan 80 mg or placebo per day; 3 years	Telmisartan significantly reduced cardiovascular death (HR,0.42; 95% CI, 0.38 to 0.61; p < 0.0001), and hospital admission of chronic heart failure (HR, 0.38; 95% CI, 0.19 to 0.51; p < 0.0001) in 3 years in patients on maintenance hemodialysis compared with placebo.

ARB, angiotensin receptor blocker; CI, confidence interval; CKD, chronic kidney disease; eGFR, estimated glomerular filtration rate; HR, hazard ratio; RAAS, renin–angiotensin–aldosterone system; RCT, randomized controlled trial

A study showed that add-on administration of 80 mg/day of telmisartan for 4–7 years did not significantly decrease cardiovascular outcomes in pre-dialysis patients with CKD (Tobe et al., 2011). Another study showed that add-on administration of 10–40 mg/day of olmesartan for 4 years did not significantly decrease cardiovascular outcomes in pre-dialysis patients with CKD (Imai et al., 2011). Contrarily, another study reported that add-on administration of 20–80 mg/day of olmesartan significantly improved cardiovascular outcomes in pre-dialysis patients with CKD during a 3-years observation period (Kim-Mitsuyama et al., 2018).

One study reported the cardioprotective effects of ARBs in on-dialysis patients with CKD (Cice et al., 2010). That study showed that add-on administration of 80 mg/day telmisartan for 3 years significantly decreased cardiovascular death and hospitalization for chronic heart failure over 3 years on-dialysis patients with CKD who had chronic heart failure (Cice et al., 2010).

**FIGURE 4:**
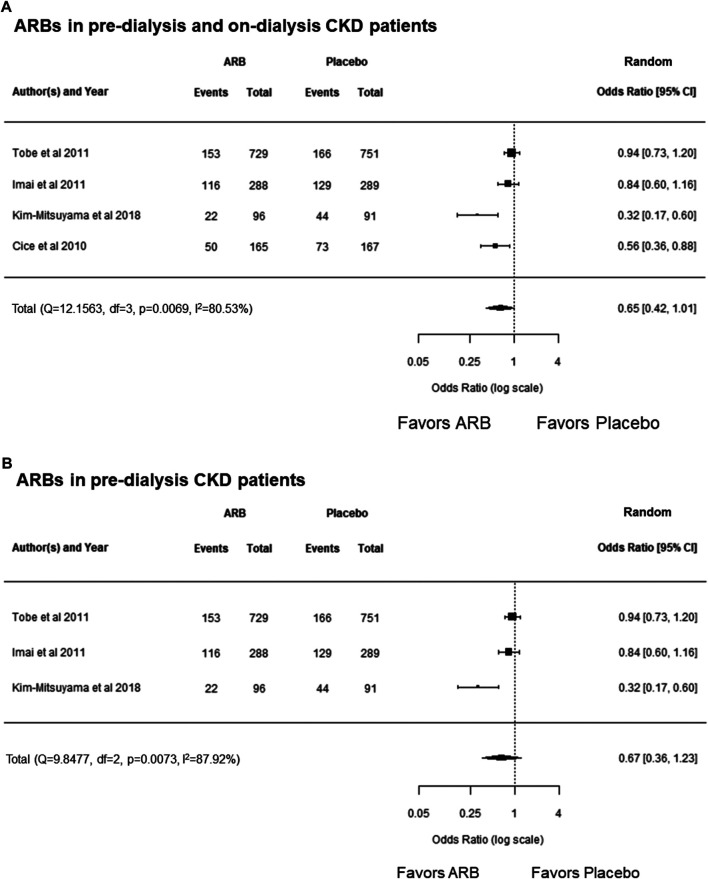
**(A)** Forest plot describing a comparison of the incidence of cardiovascular events between ARBs and placebo in pre-dialysis and on-dialysis patients with CKD. **(B)** Forest plot describing a comparison of the incidence of cardiovascular events between ARBs and placebo in pre-dialysis patients with CKD. ARB, angiotensin receptor blocker; CI, confidence intervals; CKD, chronic kidney disease.

In this study, meta-analysis revealed no significant decrease in cardiovascular events compared with the placebo group in pre-dialysis and on-dialysis CKD patients who took ARBs (OR, 0.65; 95% CI, 0.42 to 1.01; *p* = 0.0529) (Figure 4A). Additionally, meta-analysis showed no significant difference in reducing cardiovascular events in pre-dialysis patients with CKD compared with the placebo group (OR, 0.67; 95% CI, 0.36 to 1.23; *p* = 0.1936) (Figure 4B).

### Mineralocorticoid Receptor Antagonists

In the data base research in pre-dialysis patients with CKD and on-dialysis patients with CKD who took MRAs, two studies in pre-dialysis patients with CKD patients and two studies in on-dialysis patients with CKD were included in this systematic review and meta-analysis (Tsujimoto and Kajio, 2018; Lin et al., 2016; Walsh et al., 2015; Table 2). A study reported that once-daily 25 mg or 50 mg administration of eplerenone for 3 years was shown to significantly reduce the risk of cardiovascular events in pre-dialysis patients with CKD who had chronic heart failure (Eschalier et al., 2013).

**TABLE 2:** Effects of mineralocorticoid receptor antagonists for the development of cardiovascular disease in pre-dialysis or on-dialysis patients with CKD.

RAAS blocker class	Authors, Year; Reference number	Patients	Study design	Study protocol	Results
Mineralocorticoid receptor antagonists	Eschalier et al., 2013; 5	N = 912 eGFR 30 < 60 (mL/min/1.73 m^2^) Chronic heart failure with reduced ejection fraction <35 %	RCT; Multicenter double-blind placebo-controlled clinical trial	Eplerenone 25–50 mg once daily or placebo; 3 years	Compared with placebo, eplerenone reduced the risk of cardiovascular events, including hospitalization for heart failure or cardiovascular mortality, compared with placebo in patients with CKD (HR, 0.62; 95% CI, 0.49 to 0.79; p = 0.0001).
	Tsujimoto and Kajio et al., 2018; 27	N = 1465; eGFR 30 < 60 (mL/min/1.73 m^2^) or urine albumin-to-creatinine ratio >30 mg/gCre; Left ventricular ejection fraction >45%	RCT; Multicenter double-blind placebo-controlled clinical trial	Spironolactone or placebo (Dose was not shown); 6 years	Compared with placebo, spironolactone reduced cardiovascular events, including non-fatal myocardial infarction, non-fatal stroke or hospitalization for heart failure, in patients associated with CKD (HR, 0.75; 95% CI, 0.60 to 0.95; p = 0.01).
	Lin et al., 2016; 14	N = 253; Hemodialysis	RCT; Multicenter double-blind placebo-controlled clinical trial	Spironolactone 25 mg or placebo per day after hemodialysis or in the morning; 2 years	Compared with placebo, spironolactone reduced the risk of a composite death from cardiocerebrovascular events, including new occurrence or exacerbation of heart failure that was not improved by water removal through dialysis, ventricular fibrillation, or sustained ventricular tachycardia, new or recurrent acute myocardial infarction, new occurrence or exacerbation of angina pectoris, dissecting aneurysm of the aorta, stroke, and new or recurrent transient ischemic attack in patients on maintenance hemodialysis (HR, 0.42; 95% CI, 0.26 to 0.78; p = 0.017).
	Walsh et al., 2015; 28	N = 146; Dialysis, including hemodialysis and peritoneal dialysis	RCT; Multicenter double-blind placebo-controlled clinical trial; Secondary outcomes	Eplerenone 50 mg or placebo per day; 13 weeks	Compared with placebo, eplerenone did not reduce the risk of cardiovascular events in patients on maintenance hemodialysis (relative risk, 0.7; 95% CI, 0.2 to 2.3; p value was not shown.).

CI, confidence interval; CKD, chronic kidney disease; eGFR, estimated glomerular filtration rate; HR, hazard ratio; RAAS, renin–angiotensin–aldosterone system; RCT, randomized controlled trial

Another study showed that administration of spironolactone for 6 years significantly reduced cardiovascular events in pre-dialysis patients with CKD who had chronic heart failure (Tsujimoto and Kajio, 2018). The other study reported the cardioprotective effects of spironolactone in on-dialysis (hemodialysis) patients with CKD (Lin et al., 2016). That study reported that administration of 25 mg/day of spironolactone significantly reduced the risk of death from cardiocerebrovascular events in on-dialysis (hemodialysis) patients with CKD compared with the control group who were administrated placebo for the 2-years observation period (Lin et al., 2016). Contrarily, another study reported that add on administration of 50 mg/day eplerenone did not significantly decrease cardiovascular events in on-dialysis (hemodialysis and peritoneal dialysis) patients with CKD during a 13-weeks observation period (Walsh et al., 2015).

**FIGURE 5:**
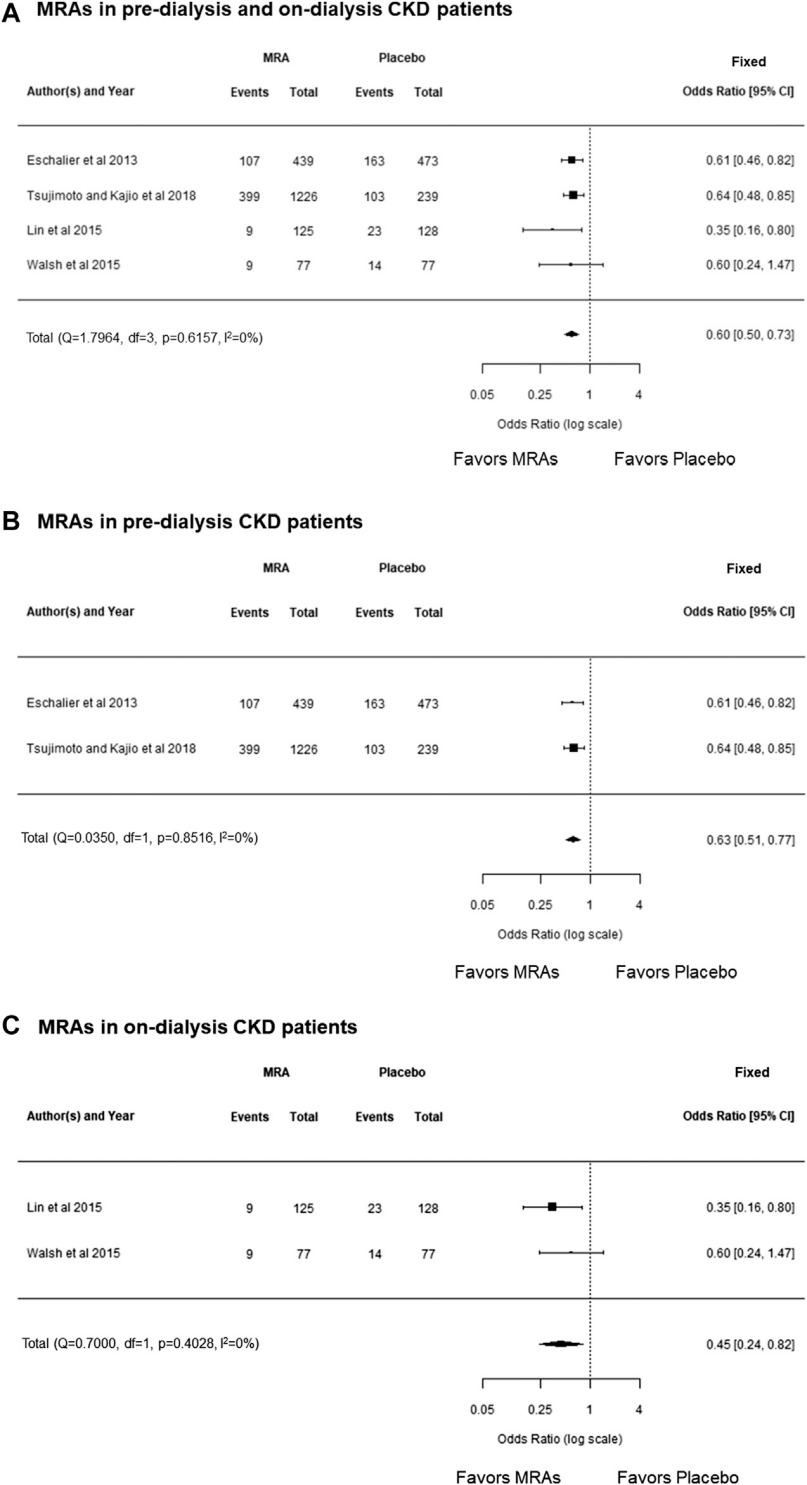
**(A)** Forest plot describing a comparison of the incidence of cardiovascular events between MRAs and placebo in pre-dialysis and on-dialysis patients with CKD. **(B)** Forest plot describing a comparison of the incidence of cardiovascular events between MRAs and placebo in pre-dialysis patients with CKD. **(C)** Forest plot describing a comparison of the incidence of cardiovascular events between MRAs and placebo in on-dialysis patients with CKD. CI, confidence intervals; CKD, chronic kidney disease; MRAs, mineralocorticoid receptor antagonists.

In this study, a meta-analysis showed that MRAs decreased cardiovascular events compared with the placebo group in both pre-dialysis and on-dialysis patients with CKD (OR, 0.60; 95% CI, 0.50 to 0.73; *p* < 0.0001), and there was no heterogeneity (*p* = 0.6157, *I* ^2^ = 0.0%) (Figure 5A). Additionally, MRAs decreased cardiovascular events compared with the placebo group in pre-dialysis patients with CKD (OR, 0.63; 95% CI, 0.51 to 0.77; *p* < 0.0001), which showed no heterogeneity (*p* = 0.8516, *I* ^2^ = 0.0%) (Figure 5B), and in the on-dialysis patients with CKD (OR, 0.45; 95% CI, 0.24 to 0.82; *p* = 0.0091), which also showed no heterogeneity (*p* = 0.4028, *I* ^2^ = 0.0%) (Figure 5C). Taken together, the results of this meta-analysis showed that MRAs showed protection effects of cardiovascular disease both in pre-dialysis patients and on-dialysis patients with CKD.

### Angiotensin Converting Enzyme Inhibitors

Only one clinical study reported the effects of ACEIs on the development of cardiovascular events in pre-dialysis patients with CKD, and the results are summarized in Table 3 (Bowling et al., 2013). Add-on administration of 2.5–20 mg/day of enalapril for 3 years significantly reduced cardiovascular hospitalization in pre-dialysis patients with CKD (Stage 1–5) who had chronic heart failure (Bowling et al., 2013). We could not perform a meta-analysis to investigate the effects of ACEIs on cardiovascular events in patients with CKD because there was only one cohort and the statistical power would have been low. Further cohorts to investigate the effects of ACEIs on cardiovascular events in patients with CKD are required to confirm the utility of ACEIs for reducing cardiovascular events in patients with CKD.

**TABLE 3:** Effects of angiotensin converting enzyme inhibitors on the development of cardiovascular disease in pre-dialysis patients with CKD.

Class of RAAS blocker	Authors, Year; Reference number	Patients	Study design	Study protocol	Results
ACEI	Bowling et al., 2013; 2	N = 1036; Stage 1–5 Chronic heart failure with ejection fraction <35% and serum creatinine <2.5 mg/dL	RCT; Multicenter double-blind placebo-controlled clinical trial	enalapril 2.5–20 mg daily or placebo; 3 years	Enalapril reduced cardiovascular hospitalization in patients with CKD compared with placebo (HR, 0.77; 95% CI, 0.66 to 0.90; p < 0.001).

ACEI, angiotensin converting enzyme inhibitor; CI, confidence interval; CKD, chronic kidney disease; HR, hazard ratio; RAAS, renin–angiotensin–aldosterone system; RCT, randomized controlled trial

### Direct Renin Inhibitors

Several studies reported that DRIs may be effective for treating hypertension in patients with CKD (Morishita et al., 2011; Sakai et al., 2012; Ito et al., 2014). However, there are no studies on the cardioprotective effects DRIs in pre-dialysis or on-dialysis patients with CKD. Therefore, we could not perform a meta-analysis on the effects of DRIs on cardiovascular events in patients with CKD.

### Combination Therapy

Two studies reported that combination therapy using ARBs and ACEIs was not effective in reducing cardiovascular events in pre-dialysis patients with CKD (Fried et al., 2013; Torres et al., 2014; Table 4). A study reported that administration of lisinopril on telmisartanl for 5–8 years did not significantly reduce cardiovascular hospitalization in pre-dialysis patients with CKD (Torres et al., 2014). Additionally, another study reported that administration of 50–100 mg of losartan on 50–100 mg of losartan and 10–40 mg of lisinopril for 4 years did not significantly reduce cardiovascular events in pre-dialysis patients with CKD (Stage 2–3) (Fried et al., 2013).

**TABLE 4:** The effects of angiotensin receptor blockers and angiotensin converting enzyme inhibitor combination therapy on the development of cardiovascular disease in pre-dialysis patients with CKD.

Class of RAAS blocker	Authors, Year; Reference number	Patients	Study design	Study protocol	Results
Combination therapy of ACEI and ARB	Torres et al., 2014; 26	N = 486; eGFR 25 < 60 (mL/min/1.73 m^2^); autosomal dominant polycystic kidney disease	RCT; Multicenter double-blind placebo-controlled clinical trial; Secondary outcomes	Combination of lisinopril and telmisartan compared with lisinopril and placebo (Dose was not shown); 5–8 years	There were no significant differences between the lisinopril–placebo group and the lisinopril–telmisartan group in the rate of hospitalization for cardiovascular disorders (2.30 events per 100 person-years and 1.28 events per 100 person-years, respectively) in patients with CKD.
	Fried et al., 2013; 7	N = 1448; Stage 2–3	RCT; Multicenter double-blind placebo-controlled clinical trial	Losartan 50–100 mg plus lisinopril 10–40 mg a day or losartan 50–100 mg plus placebo; 4 years	There was no significant difference in the rate of cardiovascular events, including myocardial infarction, stroke, and hospitalization for congestive heart failure, between the two groups in patients with CKD (HR, 0.97; 95% CI, 0.76 to 1.23; p = 0.79).

ACEI, angiotensin converting enzyme inhibitor; ARB, angiotensin receptor blocker; CI, confidence interval; CKD, chronic kidney disease; eGFR, estimated glomerular filtration rate; HR, hazard ratio; RAAS, renin–angiotensin–aldosterone system; RCT, randomized controlled trial

**FIGURE 6:**
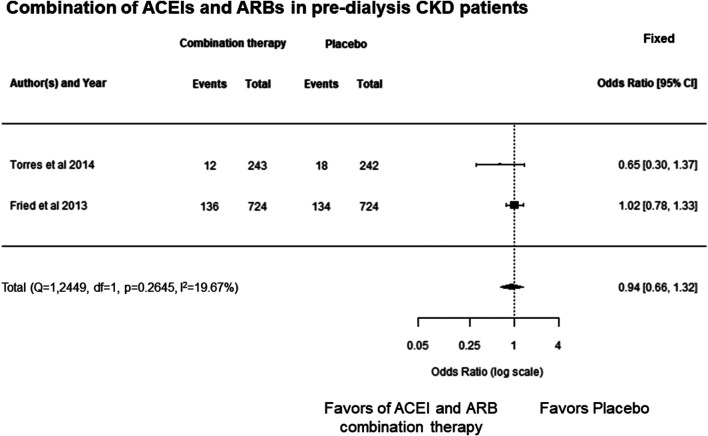
Forest plot describing a comparison of the incidence of cardiovascular events between combination therapy with ARBs and ACEIs and placebo in pre-dialysis patients with CKD. ACEIs, angiotensin converting enzyme inhibitors; ARBs, angiotensin receptor blockers; CI, confidence intervals; CKD, chronic kidney disease.

In this study, the meta-analysis showed that combination therapy with ARBs and ACEIs was not significantly different in reducing cardiovascular events compared with the placebo group in pre-dialysis or on-dialysis CKD patients (OR, 0.94; 95% CI, 0.66 to 1.32; *p* = 0.7069) and there was no heterogeneity (*p* = 0.2645, *I* ^2^ = 19.67%) (Figure 6). These results suggests that combination therapy using ARBs and ACEIs may not be effective at decreasing cardiovascular events in pre-dialysis patients with CKD. There are no studies on the cardioprotective effects of RAAS blockers used in combination in on-dialysis patients with CKD.

## Discussion

In this study, the meta-analysis showed that RAAS blockers (ARBs, ACEIs, MRAs, and combination of ARBs and ACEIs) significantly decreased cardiovascular events compared with the placebo group in on-dialysis patients with CKD. However, those effects could not be shown in pre-dialysis patients with CKD owing to heterogeneity among the cohorts (Figures 2A–C). Additionally, we found significant publication bias that the studies showing beneficial effects of RAAS blockers for protection of cardiovascular disease in patients with CKD (Figure 3). That results also may support careful estimations of the beneficial effects of RAAS blocker for prevention of cardiovascular disease in patient with CKD.

In each class of RAAS blockers, meta-analysis revealed MRAs decreased cardiovascular events compared with the placebo group in pre-dialysis and on-dialysis patients with CKD (Figures 5A–C); however, ARBs and the combination of ARBs and ACEIs were failed to show decrease cardiovascular events in those populations determined by range of 95% CI and heterogeneity among the cohorts (Figures 4A,B; Figure 6). These results may suggest MRA may have beneficial effects for decreasing cardiovascular events in pre-dialysis and on-dialysis patients with CKD. In this study, the effects of ACEIs and DRIs for cardiovascular diseases in patients with CKD could not be analyzed owing to lack of study number. Additionally, it should be note that heterogeneity among cohorts and possible publication bias (Figure 3) affected the results of this study. Therefore, further randomized controlled studies will need to investigate the effects of RAAS blockers for cardiovascular disease in patients with CKD.

Our systematic review and meta-analysis have several limitations. First, we only searched for studies that were published in English. Second, we only used the PubMed database to identify publications. Third, cardiovascular outcomes were different among studies. For example, one study included stroke as a cardiovascular event (Tsujimoto and Kajio, 2018), while another study did not include stroke as a cardiovascular event (Cice et al., 2010). Fourth, the random-effects model was used in the outcome analyses because of the high heterogeneity, which may be related to different doses and intervention duration of the RAAS blockers. Therefore, studies that are designed as high-quality, large-scale randomized controlled trials are required to evaluate the effectiveness of RAAS blockers to protect against the development of cardiovascular disease in patients with CKD.

In conclusion, RAAS blockers significantly reduced cardiovascular events in on-dialysis patients with CKD, but there were no significant results in pre-dialysis patients with CKD because of the heterogeneity in each study. Among the RAAS blockers, MRAs may decrease cardiovascular events in pre-dialysis and on-dialysis patients with CKD. However, other RAAS blockers, such as ARBs, ACEIs, and DRIs, did not show these cardioprotective effects in these populations. This was at least partially because of the small number of cohorts. Therefore, additional large-scale cohorts are required to investigate the effects of RAAS blockers on cardiovascular disease in patients with CKD.

## Data Availability

The original contributions presented in the study are included in the article/Supplementary Material, further inquiries can be directed to the corresponding author.
