# Supplementary material for: Systematic Review and Meta-Analysis of Renin–Angiotensin–Aldosterone System Blocker Effects on the Development of Cardiovascular Disease in Patients With Chronic Kidney Disease
Source: Front Pharmacol. 2021 Jul 2;12:662544. doi: 10.3389/fphar.2021.662544 (PMC8283791; doi:10.3389/fphar.2021.662544)
Supplement: Supplementary file 1 [file Table1.docx]

| Supplemental Table 1. Full search results | |  |
| --- | --- | --- |
| Database | Query | No of records |
| PubMed | "renin angiotensin aldosterone AND cardiovascular disease AND chronic kidney disease" | 35 |
|  | "renin angiotensin aldosterone AND heart failure AND chronic kidney disease" | 16 |
|  | "renin angiotensin aldosterone AND heart attack AND chronic kidney disease" | 5 |
|  | "renin angiotensin aldosterone AND cardiovascular disease AND hemodialysis" | 9 |
|  | "renin angiotensin aldosterone AND heart failure AND hemodialysis" | 4 |
|  | "renin angiotensin aldosterone AND heart attack AND hemodialysis" | 3 |
|  | "renin angiotensin aldosterone AND cardiovascular disease AND peritoneal dialysis" | 0 |
|  | "renin angiotensin aldosterone AND heart failure AND peritoneal dialysis" | 0 |
|  | "renin angiotensin aldosterone AND heart attack AND peritoneal dialysis" | 0 |
|  | "ARB AND cardiovascular disease AND chronic kidney disease" | 32 |
|  | "ARB AND heart failure AND chronic kidney disease" | 8 |
|  | "ARB AND heart attack AND chronic kidney disease" | 5 |
|  | "ARB AND cardiovascular disease AND hemodialysis" | 12 |
|  | "ARB AND heart failure AND hemodialysis" | 3 |
|  | "ARB AND heart attack AND hemodialysis" | 4 |
|  | "ARB AND cardiovascular disease AND peritoneal dialysis" | 0 |
|  | "ARB AND heart failure AND peritoneal dialysis" | 0 |
|  | "ARB AND heart attack AND peritoneal dialysis" | 0 |
|  | "angiotensin receptor blocker AND cardiovascular disease AND chronic kidney disease" | 87 |
|  | "angiotensin receptor blocker AND heart failure AND chronic kidney disease" | 28 |
|  | "angiotensin receptor blocker AND heart attack AND chronic kidney disease" | 14 |
|  | "angiotensin receptor blocker AND cardiovascular disease AND hemodialysis" | 24 |
|  | "angiotensin receptor blocker AND heart failure AND hemodialysis" | 10 |
|  | "angiotensin receptor blocker AND heart attack AND hemodialysis" | 7 |
|  | "angiotensin receptor blocker AND cardiovascular disease AND peritoneal dialysis" | 1 |
|  | "angiotensin receptor blocker AND heart failure AND peritoneal dialysis" | 1 |
|  | "angiotensin receptor blocker AND heart attack AND peritoneal dialysis" | 0 |
|  | "ACEI AND cardiovascular disease AND chronic kidney disease" | 15 |
|  | "ACEI AND heart failure AND chronic kidney disease" | 7 |
|  | "ACEI AND heart attack AND chronic kidney disease" | 4 |
|  | "ACEI AND cardiovascular disease AND hemodialysis" | 7 |
|  | "ACEI AND heart failure AND hemodialysis" | 3 |
|  | "ACEI AND heart attack AND hemodialysis" | 2 |
|  | "ACEI AND cardiovascular disease AND peritoneal dialysis" | 0 |
|  | "ACEI AND heart failure AND peritoneal dialysis" | 0 |
|  | "ACEI AND heart attack AND peritoneal dialysis" | 0 |
|  | "angiotensin converting enzyme inhibitor AND cardiovascular disease AND chronic kidney disease" | 83 |
|  | "angiotensin converting enzyme inhibitor AND heart failure AND chronic kidney disease" | 38 |
|  | "angiotensin converting enzyme inhibitor AND heart attack AND chronic kidney disease" | 21 |
|  | "angiotensin converting enzyme inhibitor AND cardiovascular disease AND hemodialysis" | 26 |
|  | "angiotensin converting enzyme inhibitor AND heart failure AND hemodialysis" | 10 |
|  | "angiotensin converting enzyme inhibitor AND heart attack AND hemodialysis" | 9 |
|  | "angiotensin converting enzyme inhibitor AND cardiovascular disease AND peritoneal dialysis" | 1 |
|  | "angiotensin converting enzyme inhibitor AND heart failure AND peritoneal dialysis" | 1 |
|  | "angiotensin converting enzyme inhibitor AND heart attack AND peritoneal dialysis" | 0 |
|  | "direct renin inhibitor AND cardiovascular disease AND chronic kidney disease" | 5 |
|  | "direct renin inhibitor AND heart failure AND chronic kidney disease" | 1 |
|  | "direct renin inhibitor AND heart attack AND chronic kidney disease" | 1 |
|  | "direct renin inhibitor AND cardiovascular disease AND hemodialysis" | 4 |
|  | "direct renin inhibitor AND heart failure AND hemodialysis" | 1 |
|  | "direct renin inhibitor AND heart attack AND hemodialysis" | 1 |
|  | "direct renin inhibitor AND cardiovascular disease AND peritoneal dialysis" | 0 |
|  | "direct renin inhibitor AND heart failure AND peritoneal dialysis" | 0 |
|  | "direct renin inhibitor AND heart attack AND peritoneal dialysis" | 0 |
|  | "mineralocorticoid receptor antagonist AND cardiovascular disease AND chronic kidney disease" | 39 |
|  | "mineralocorticoid receptor antagonist AND heart failure AND chronic kidney disease" | 25 |
|  | "mineralocorticoid receptor antagonist AND heart attack AND chronic kidney disease" | 2 |
|  | "mineralocorticoid receptor antagonist AND cardiovascular disease AND hemodialysis" | 11 |
|  | "mineralocorticoid receptor antagonist AND heart failure AND hemodialysis" | 7 |
|  | "mineralocorticoid receptor antagonist AND heart attack AND hemodialysis" | 1 |
|  | "mineralocorticoid receptor antagonist AND cardiovascular disease AND peritoneal dialysis" | 2 |
|  | "mineralocorticoid receptor antagonist AND heart failure AND peritoneal dialysis" | 2 |
|  | "mineralocorticoid receptor antagonist AND heart attack AND peritoneal dialysis" | 0 |
|  |  | Total 637 |
